# Dr. H. J. McKellops

**Published:** 1901-09

**Authors:** 


### Obituary.

### DR. H. J. McKELLOPS.

In the death of Dr. H. J. McKellops, on April 23, 1901, St. Louis has lost the last of her pioneers in the profession of dentistry.

Dr. McKellops began the practice of the profession when scarcely half a dozen dentists had offices in St. Louis, his colleagues then being such men as Isaiah Forbes, Aaron Blake, Isaac

Comstock, J. L. Clark, C. W. Spalding, and H. E. Peebles. All of these had passed away when death closed the career of Dr. McKellops. He had been for fifty-six years an active practitioner. In that time his reputation had extended over the United States, he had contributed much to the material advancement of the profession of dentistry, and, more than any one else in the city, perhaps, had been instrumental in bringing to it the prestige it enjoys as one of the learned professions.

Dr. McKellops was born at Saline, near Syracuse, N. Y., on August 31, 1823. His father, James McKellops, died before his son had entered his teens. In 1840 he came to St. Louis with his mother and sister, entering one of the public schools. Active and intelligent, he soon after obtained appointment as a messenger in the Missouri Legislature, using the money thus earned for tuition at the University of Missouri, at Columbia, where he studied from 1842 to 1844. A course of book-keeping in Jones's Commercial College followed this schooling, after which he found employment in the City Register's office, where opportunity presented for forming a wide circle of acquaintances, valuable afterwards when he entered the practice of dentistry.

He was drawn to the study of medicine in 1846 and 1847, at the old St. Louis Medical College, of which Dr. Charles A. Pope was Dean. For the next six years he attended many of the lectures, but never took the degree of M.D.

Through the persuasion of his brother-in-law, Dr. George Silvers, a dentist, he was drawn from medicine to the practice of dentistry. Natural ingenuity and a love of the mechanical arts soon made him an expert operator, and soon after opening his first office he commanded a practice of the highest class.

In 1855 the degree of D.D.S. was conferred on him by the Ohio College of Dental Surgery, in recognition of his skill and services to the profession, his fame already having extended through Missouri, and eventually through all the States of the Union. Dr. McKellops introduced in St. Louis the use of continuous gum work, invented by Dr. John Allen, of New York. In his profession he was studious, inventive in practice, and always to the front in every step of progress in dental surgery.

A loving-cup was left by him on which appears the following: "Presented to Dr. H. J. McKellops by the First Dental Society of the State of New York, as an expression of the high esteem and as a token of its appreciation of the inestimable services rendered by him as Supervisor of Clinics at the Annual Meetings, New York City, January 21, 1891."

The St. Louis Dental Society, on March 24, 1900, tendered Dr. McKellops a banquet as a token of esteem and love.

Personally Dr. McKellops was a man of high ideals, sociable and warm-hearted. His sociability found expression in the field of his profession, in the organization of dental societies and associations, in the proceedings of which he always took a leading part. He was one of the organizers of the St. Louis Dental Society, founded on December 9, 1856, and in 1879 served as its president. He helped organize also the Western Dental Association in 1860, was first president of the Missouri State Dental Association in 1865, served as president of the American Dental Association in 1878, and in 1884 was elected president of the Southern Dental Association.

In social life, no less than in his profession, Dr. McKellops was popular, and he was a particularly welcome guest at social gatherings, because of his high intelligence and brilliant powers of entertaining.

The observations of the necessity of dentists in the army no doubt prompted Dr. McKellops to introduce a resolution at a meeting of the Western Dental Society, held in Quincy, Ill., on July 21, 1858, to the effect that a committee be appointed to memorialize Congress on the necessity of appointing dentists to be attached to the regular army. The resolution was adopted, and a similar resolution was passed by the American Dental Convention in August the same year, being introduced by Dr. McKellops.

Dr. McKellops married Miss Annie Gower, of Tennessee, on April 1, 1849. Eight children were born,—five sons and three daughters. Those now living are Dr. Henry L. McKellops and Mrs. Josephine Bouvier, of San Francisco, Linton J. McKellops and Dr. Leo C. McKellops, of St. Louis, and Gerald G. McKellops, of Cincinnati.

John G. Harper,
Burton L. Thorpe,
Edward H. Angle,
*Committee.*

